# A neural signature of contextually mediated intentional forgetting

**DOI:** 10.3758/s13423-016-1024-7

**Published:** 2016-05-05

**Authors:** Jeremy R. Manning, Justin C. Hulbert, Jamal Williams, Luis Piloto, Lili Sahakyan, Kenneth A. Norman

**Affiliations:** 1 Department of Psychological and Brain Sciences, Dartmouth College, Hanover, NH 03755 USA; 2 Psychology Program, Bard College, Annandale-on-Hudson, NY 12504 USA; 3 Psychology Department, University of Illinois at Urbana-Champaign, Champaign, IL 61801 USA; 4 Princeton Neuroscience Institute, Princeton University, Princeton, NJ 08540 USA; 5 Department of Psychology, Princeton University, Princeton, NJ 08540 USA

**Keywords:** Directed forgetting, Context, Episodic memory, fMRI

## Abstract

The *mental context* in which we experience an event plays a fundamental role in how we organize our memories of an event (e.g. in relation to other events) and, in turn, how we retrieve those memories later. Because we use contextual representations to retrieve information pertaining to our past, processes that alter our representations of context can enhance or diminish our capacity to retrieve particular memories. We designed a functional magnetic resonance imaging (fMRI) experiment to test the hypothesis that people can *intentionally* forget previously experienced events by changing their mental representations of contextual information associated with those events. We had human participants study two lists of words, manipulating whether they were told to forget (or remember) the first list prior to studying the second list. We used pattern classifiers to track neural patterns that reflected contextual information associated with the first list and found that, consistent with the notion of contextual change, the activation of the first-list contextual representation was lower following a forget instruction than a remember instruction. Further, the magnitude of this neural signature of contextual change was negatively correlated with participants’ abilities to later recall items from the first list.

## Introduction

Although it is frustrating when we forget information unintentionally, sometimes forgetting can be beneficial. Here we ask *how* we can intentionally forget recently experienced events by examining neuroimaging data collected during a *list-method directed forgetting* experiment. This paradigm asks participants to forget list A items prior to studying list B, resulting in impaired list A recall and enhanced list B recall (Bjork et al. 1968; Basden et al. 1993; Johnson 1994; MacLeod 1998; Sahakyan et al. 2013).

The most prevalent explanation for degraded list A recall following a forget instruction is that participants change their mental context (Sahakyan and Kelley 2002), possibly using executive control mechanisms (Anderson and Hanslmayr 2014). According to this view, list A items are associated with whatever other thoughts are present at the time they are studied; these co-active thoughts constitute the participant’s *mental context* (Manning et al. 2014), and can be internally generated (e.g. spontaneous thoughts) or externally generated (e.g. thoughts about the immediate sensory experience). Normally, these mental context representations persist until the recall test, at which point they can facilitate list A recall. However, when participants receive a forget cue, they deliberately change their mental context, such that previously active contextual elements become less active, and (possibly) new contextual elements become active. Consequently, the contextual features that were (previously) linked to list A items are no longer available at test, impairing list A recall. Note that the *benefits* of directed forgetting (i.e., enhanced list B recall) are thought to result primarily from mechanisms other than context change (Sahakyan et al. 2013). Our focus in this paper is on explaining the *costs* of directed forgetting (i.e., impaired list A recall).

While behavioral data support the contextual change hypothesis (Sahakyan and Kelley 2002; Sahakyan et al. 2013), no study to date has obtained *neural* evidence for the hypothesis. Here we used multivariate pattern analysis (MVPA) of fMRI data to track context-related neural patterns as participants studied word lists A and B. We presented images (passively viewed by the participants) of outdoor scenes interspersed between the list A words, but not the list B words. Our goal was to inject scene information into participants’ mental contexts during list A study and then to assess (using MVPA) the extent to which scene information persisted during later points in the experiment, when scenes were not displayed. We interpreted high levels of persistent scene activity after list A as reflecting continued activation of the list A context, an approach we have employed successfully in another context-dependent memory paradigm (Gershman et al. 2013). We hypothesized that if participants change their mental context after forget cues, we should see a corresponding decrease in scene activity following the forget cue. Further, we hypothesized that the size of this decrease should negatively correlate with participants’ list A recall performance.

## Methods

### Participants

Our functional neuroimaging study at Princeton University included 25 participants (ten male, 15 female), ages 19 to 34 (mean ± SEM: 21.3 ± 0.6 years). We excluded one 19-year-old male participant from all of our analyses because his recall performance was near perfect (and greater than 3 standard deviations above the mean performance) in all of the experimental conditions (see *Experimental paradigm*), which limited our ability to observe his behavioral and neural signatures of intentional forgetting. The participants were paid $20/hour for their participation, and experimental testing sessions lasted approximately 1.5 hours. Our experimental protocol was approved by Princeton’s institutional review board.

Our sample size was chosen a priori based on sample sizes used in previous list-method directed forgetting studies (Sahakyan et al. 2013). Accounting for differences in our experimental design (whereby each participant in our experiment experienced multiple experimental conditions), we estimated that a sample size of between 20 and 30 participants would provide a reasonable test of our main hypothesis.

### Experimental paradigm

Our experimental paradigm was organized into eight *study-test* blocks and one *localizer* block. Each of these blocks occurred during a distinct functional run (see *Functional neuroimaging*).

**Fig. 1:**
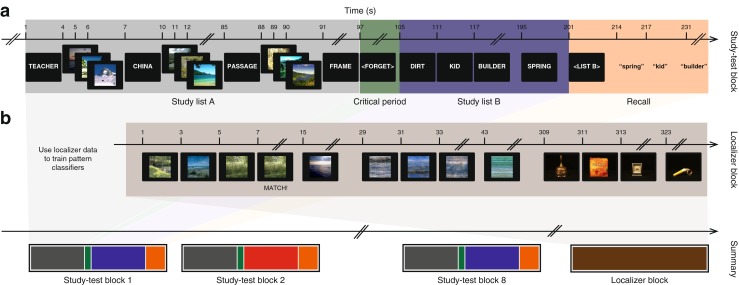
Methods overview. **a** Study-test block. During each study-test block, participants first studied list A while passively viewing images of outdoor scenes between the word presentations. They then received a memory cue instructing them to either forget or remember the list A words. Next they studied list B (without viewing scene images between the word presentations). Finally, they were instructed to verbally recall as many words (from either list A or list B) as they could remember in 1 min. Participants experienced a total of eight study-test blocks during the experiment. **b** Localizer block. During each localizer block, participants viewed images drawn from three categories: outdoor scenes, phase-scrambled scenes, and everyday objects. Participants were instructed to press a button on a handheld control pad when an image exactly matched the image that preceded it. Data from this block were used to train pattern classifiers to estimate scene-related activity during the study-test blocks. A summary of the entire experiment, comprising eight study-test blocks and the localizer block, is shown at the bottom of the figure; *blue* indicates list B study following a forget cue, and *red* indicates list B study following a remember cue

In each *study-test* block (Fig. 1a), participants viewed a central fixation cross for 3 s (not shown in the figure), followed by a 3-s delay. They then studied a 16-word list, list A, followed by an on-screen *memory cue* instruction telling them to either *forget* or *remember* the list A items. Participants then studied a second 16-word list, list B. (See *List construction* for a detailed description of how we generated the random word lists.) Finally, participants received an on-screen *recall cue* instructing them to verbally recall either list A or list B (they were given 1 minute to recall the words in any order they wished). Prior to the start of the experiment, participants were told that, with 100% certainty, a forget instruction meant that they would be asked to recall list B on that block (despite this instruction, we tested participants’ memory for list A on the final forget block as described below). We also told participants (truthfully) that if they instead received a remember instruction, they would be asked to recall either list A or list B, with equal probability.

Each list word appeared onscreen for 3 s, and the word presentations were separated by 3 s. During the 3-s inter-word intervals between list A words, participants viewed three randomly chosen images of outdoor scenes (presented for 1 s each in immediate succession). Crucially, scenes were not presented during the inter-word intervals during list B study (instead, the screen was left blank). Each scene image appeared only once during the entire experiment. Prior to the start of the experiment we (truthfully) told participants that we would not test their memory for the scene images, but that they should passively view the scene images when they appeared.

Each participant received a total of four remember instructions and four forget instructions, across the eight study-test blocks they experienced. The order in which participants experienced remember or forget blocks was randomized independently for each participant, subject to the constraints that (a) the same cue type could not appear in 3 successive blocks, and (b) the last block was always a forget block. (These two constraints also meant that the second-to-last block was always a remember block.) During two (randomly chosen) remember blocks, participants were asked to recall the list A words, and on the remaining two remember blocks the participants were asked to recall the list B words. Participants were asked to recall list B words on every forget block except the last, when they were instead asked to recall the list A words. In other words, participants were misled into believing they could forget the list A words during the last study-test block, but were then nonetheless asked to recall list A. This allowed us to study the behavioral and neural effects of the forget instruction on the to-be-forgotten information.

The localizer block (Fig. 1b) occurred after the last study-test block. The localizer block provided data for training pattern classifiers to track scene-related activity throughout the study-test blocks. In this block, participants viewed images from three categories: outdoor natural scenes, Fourier phase-scrambled images of outdoor natural scenes (where each color channel was scrambled independently and then re-combined), and everyday objects. The outdoor natural scene images used in our experiment were selected from the Scene UNderstanding (SUN) Database (Xiao et al. 2010) and the object images were selected from the Amsterdam Library of Object Images (ALOI; Geusebroek et al., 2005). Each image was displayed for 500 ms followed by a 1500-ms pause. Images were organized into 27 sets of eight same-category images (nine sets per category; the assignment of images to sets was done randomly for each participant). Each set of eight images was displayed (one at a time), followed by a 12-s pause before the next set of eight. Participants performed a *one-back task* as they viewed the images, whereby they were instructed to press a button on a handheld controller when an image exactly matched the image that preceded it. (Repetitions occurred on 15% of the image presentations.)

#### List construction

Each participant studied a total of sixteen 16-word lists (2 per block). All of the participants studied the same lists, but in a unique randomized order. Each list was assigned (randomly for each participant) to one of the four experimental conditions. To construct the lists, we first drew 256 words uniformly at random from the Medical Research Council Psycholinguistic Database (Coltheart 1981). We then constructed 16 lists that were matched according to word frequency (mean 49.3, SD 16.9), number of letters (mean 5.4, SD 0.3), number of syllables (mean 1.7, SD 0.1), concreteness (mean 540.3, SD 12.0), and imageability (mean 559.7, SD 8.9). (These means and standard deviations are computed across lists.)

#### Audio recording

We recorded participants’ verbal recalls using a customized MR-compatible recording system (FOMRI II, Optoacoustics Ltd.). We used the Penn TotalRecall tool (http://memory.psych.upenn.edu) to score and annotate the verbal responses.

### Functional neuroimaging

#### Imaging parameters

All participants were scanned using a Siemens Skyra 3-T full-body scanner (Siemens, Erlangen, Germany) with a volume head coil. We collected, from each participant, ten functional runs: eight study-test blocks and one localizer block (see *Experimental paradigm*), plus one additional run (in which participants studied and recalled a list of 12 words prior to the localizer block) that we did not examine in this paper (it was not relevant to the current paradigm or the analyses presented here). The functional runs comprised T2*-weighted gradient-echo echo-planar (EPI) sequences (voxel size =3×3×3 mm; repetition time [TR] = 2000 ms, echo time [TE] = 30 ms; flip angle =71^∘^; matrix =64×64; slices = 36; field of view [FoV] = 192 mm). We also collected, for each participant, a single high-resolution T1-weighted magnetization-prepared rapid-acquisition gradient echo (MPRAGE) image to facilitate registration and normalization (voxel size =1×1×1 mm; TE = 3.3 ms; flip angle =7^∘^; matrix =256×256; slices = 176; FoV = 256 mm), and a single fast low-angle shot (FLASH) field map to correct spatial distortions of the EPI images (voxel size =0.75×0.75×3 mm; TE = 2.6 ms; flip angle =70^∘^; matrix =256×256; slices = 36; FoV = 192 mm).

#### Image preprocessing

We preprocessed the fMRI data using the FMRI Expert Analysis Tool (FEAT) Version 6.00, which is part of FMRIB’s Software Library (FSL, http://www.fmrib.ox.ac.uk/fsl). We removed the first three brain volumes from each functional run to allow for T1 stabilization. We then applied the following pre-statistics processing steps to the functional images: motion correction using MCFLIRT (Jenkinson et al. 2002); slice-timing correction using Fourier-space time-series phase-shifting; non-brain removal using BET (Smith 2002); grand-mean intensity normalization of the entire 4D dataset by a single multiplicative factor; and high-pass temporal filtering (Gaussian-weighted least-squares straight line fitting, with sigma = 64.0 s). We then used FLIRT (Jenkinson et al. 2002) to register each participant’s functional images to standard (MNI) space.

#### Multivariate pattern analysis (MVPA)

After pre-processing the fMRI data, we used the Harvard-Oxford cortical atlas (Desikan et al. 2006) to define a mask (using an inclusion threshold of 25%) consisting of the union of the posterior and anterior parahippocampal gyrus; the posterior cingulate; and the anterior temporal, posterior temporal, and temporal occipital fusiform (this mask was intended to encompass the parahippocampal place area and retrosplenial cortex, as these regions have been previously implicated in scene processing; Epstein et al., 1999). We used the in-mask voxels to train L2-regularized multinomial logistic regression classifiers using data from each participant’s localizer block. (We trained the classifiers independently for each participant.) To account for the 6-s delay in the peak of the hemodynamic response function, we shifted the event labels forward in time by 6 s (three images), such that each brain volume was matched up with the event that occurred 6 s earlier. The multinomial classifiers were trained to discriminate when the participants were viewing images of scenes versus everyday objects versus phase-scrambled scenes versus rest (where “rest” was defined as the last three volumes collected during the 12-s pause between the eight-image blocks). We evaluated the classifiers’ abilities to estimate scene-related activity (versus non-scene activity) using ninefold cross-validation applied to data from the localizer block (mean area under the receiver operating characteristic curves across 24 participants ± SEM: 0.78 ± 0.006). We used the trained classifiers to predict the degree of scene-related activity (ranging from 0 to 1, inclusive) reflected in each brain volume collected during the study-test blocks.

The primary goal of our study was to test the hypothesis that participants respond to the forget cue by changing their mental context (which we expected, at the time of the cue, to include thoughts about the scene images we presented between the list A words). This would manifest as a larger decrease in scene-related activity following a forget cue than following a remember cue. We predicted that this contextual change process would occur directly in response to the forget cue, even before participants began to study list B. We refer to this time interval (from the time of the forget/remember instruction until the beginning of list B) as the *critical period* (Fig. 1a). We defined a measure called *scene drop* to quantify the degree of contextual change following a memory cue. Scene drop was defined as the decrease in scene-related activity from just before the critical period to the time after the critical period. Specifically, for each block, we took a pre-critical-period measurement of scene activity (averaging over the interval beginning after the last scene had been presented in list A and ending just before the forget/remember memory cue) and subtracted out a post-critical-period measurement of scene activity (averaging over the interval beginning when the first word in list B appeared onscreen and ending when the last word in list B disappeared from the screen). Note that, as described above, we applied a 6-s shift in matching up scene activity estimates to events (so, for example, the first scene activity estimate assigned to the post-critical period was acquired 6 s after the beginning of list B study). Importantly, we hypothesized that scene activity could decrease from list A to list B for two reasons: (1) because scenes are no longer being viewed onscreen (this is true for both the remember and forget conditions), and (2) because participants change their mental context in the forget condition. By taking our initial measurement of scene activity *after* the last of the list A scenes was presented, we hoped to minimize the influence of the former factor (i.e., whether or not participants were actually viewing scenes) and, consequently, to increase the sensitivity of our scene drop measure to the contextual change process. We note, however, that our measure of scene drop likely included some lingering traces of scene-related activity from the list A scene presentations, which would add noise to our scene drop measure. Crucially, this activity should not exert a systematic bias on our analyses because it should be equally present, on average, in the forget and remember conditions.

## Results

**Fig. 2:**
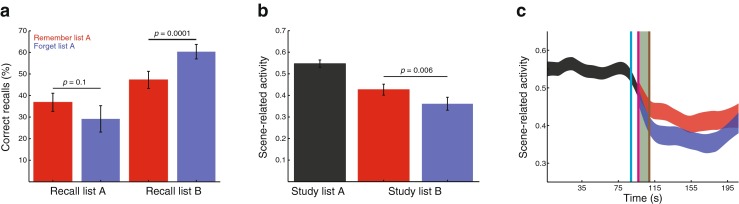
Identifying a neural signature of intentional forgetting. **a** Memory performance. The proportion of correctly recalled words is shown for each of the four experimental conditions. **b** Average scene activity. The *bars* display the mean level of scene-related activity measured by the classifier as participants studied lists A and B (the mean is taken across blocks and participants, and reflects the entire study period for each list). The list B blocks are divided according to whether participants received a forget cue (*blue*) or a remember cue (*red*) following list A. The *p* values in panels **a** and **b** are from two-tailed across-participant paired *t* tests. **c** Time course of scene-related activity. The ribbon plots display the mean level of scene-related activity measured by the classifier as each brain volume was collected (where the mean is taken across participants). The *vertical lines* denote the times of experimental events (shifted forward by 6 s to account for hemodynamic lag): the time the last scene disappeared from the screen during list A (*cyan*), the time the memory cue appeared on the screen (*magenta*), and the time the first list B word appeared on the screen (*brown*). The *green shading* denotes the critical period (Fig. 1a). *Error bars* in panels **a** and **b** and line thicknesses in panel **c** denote ±1 SEM, taken across the 24 participants

Figure 2a shows behavioral recall performance for list A and list B, as a function of whether participants were given a forget or remember cue. Specifically, we asked whether participants were better able to recall list B items following a forget cue (relative to following a remember cue) and whether they were better able to recall list A items following a remember cue (relative to following a forget cue). As described in the *Introduction*, these patterns reflect the benefit and cost of directed forgetting, respectively. Qualitatively, we observed the standard benefits and costs of directed forgetting. The benefit was significant, *t*(23)=4.6,*p*=0.0001, and the cost was trending towards significance, *t*(23)=−1.7,*p*=0.1 (note that we had a clear directional hypothesis here, and the corresponding one-tailed *p*-value is 0.05). Note that our experiment only yields a single measurement of list A recall performance after a forget cue (on the very last block); as such, we expect that any measurements involving list A recall on forget trials will be noisy.

### List A learning

We designed our study to test the hypothesis that participants respond to the forget instruction by changing their mental context. By presenting images of scenes interspersed between the list A words, we hoped that neural patterns that reflected those scenes would be (passively) incorporated into participants’ list A contextual representations. If so, the level of scene-related neural activity throughout the remainder of the experiment (as estimated by pattern classifiers trained on data from a different functional run; see *Multivariate pattern analysis*) should reflect currently active traces of list A context. Supporting the hypothesis that participants change their mental context following a forget cue, we found that participants exhibited substantially lower levels of scene activity during list B following a forget cue than following a remember cue (*t*(23)=−3.0,*p*=0.006; Fig. 2b).

Although the order in which participants experienced the experimental conditions was randomized with respect to memory cue and recall instruction for the first six study-test blocks, study-test blocks seven and eight were always a remember and forget block, respectively, and participants always recalled list A in the final block. To ensure that the observed difference in scene activity during list B was not due to order confounds, we carried out a control analysis to verify that the forget versus remember scene activity difference shown in Fig. 2 also held for the first six study-test blocks alone; this was indeed the case (scene-related activity during list B following a forget versus remember cue: *t*(23)=−3.0,*p*=0.006).

**Fig. 3:**
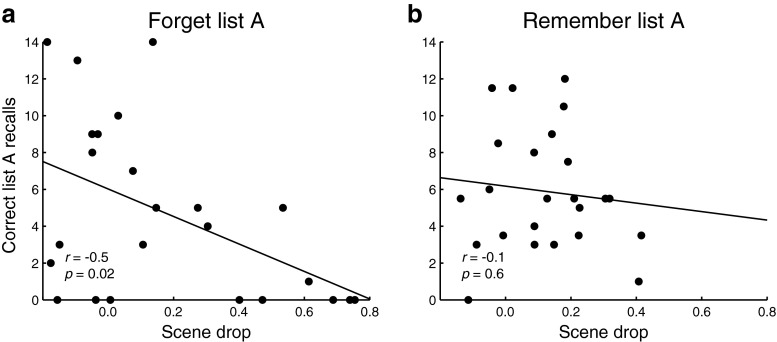
Contextual change leads to fewer list A recalls on forget blocks. **a** Forget list A blocks. The *x*-coordinate of each *dot* indicates, for a single participant, the degree of contextual change [as measured by the scene drop (see *Multivariate pattern analysis*), for the (single) block where the participant was told to forget list A and then nonetheless was asked to recall list A]. The dots’ *y*-coordinates reflect the numbers of list A items the participants recalled during those blocks. **b** Recall list A blocks. This panel is in the same format as Panel a, but reflects blocks on which participants were asked to remember list A and then asked to later recall list A. (Each *dot* reflects an average across all remember/recall list A blocks for one participant.) The correlations reported in each panel are computed across participants

If participants use currently active contextual representations to probe their memory, then the degree to which participants change their mental context should be negatively correlated with their ability to recall list A items (when asked to do so). In principle, this relationship should be present after both forget and remember cues. However, we expected that the relationship between scene drop and list A recall would be *easiest to observe* on forget blocks, where the contextual change process is hypothesized to occur. On remember blocks, if participants are not actively changing their contextual representations, there may not be enough variance to detect a relationship between scene drop and list A recall. In keeping with these ideas, we found that participants exhibited significantly greater variability in scene drop following forget (versus remember) cues (*F*(23,23)=3.96,*p*=0.002). Also, scene drop and the number of list A recalls were reliably correlated (across participants) following a forget cue (Fig. 3a; *r*=−0.5,*p*=0.02), but not following a remember cue (Fig. 3b; *r*=−0.1,*p*=0.6). We note that our finding that the correlation is significant for forget blocks but not remember blocks does not imply that the correlations differ (statistically) in strength.

By showing that participants exhibited lower levels of scene-related activity following a forget cue, and that the magnitude of scene drop was negatively correlated with participants’ list A recall performance, the above analyses provide neural support for the contextual change account of list-method directed forgetting. The image-by-image scene activity estimates (obtained every 2 s) also provide a means of observing the detailed timecourse of the response to the memory cue after list A (Fig. 2c). We observed that the scene activations were relatively high as participants studied list A words (and passively viewed scene images between the word presentations), and then began to decrease following the last scene presentation. The levels of scene-related activity began to diverge (according to which memory cue participants had received on that block) just after the cue’s appearance on the screen, after accounting for the 6-s lag in the peak of the canonical hemodynamic response function. The scene activation traces leveled off just prior to the start of list B, running roughly parallel to each other as participants studied list B. The timecourse suggests that participants change their mental context during the *critical period* beginning just after the forget cue appears and ending just prior to the start of list B.

### List B learning

When participants change their mental context following a forget cue, how is list B learning affected? Specifically, does reducing the activation of list A contextual information lead to better recall of list B? To test this possibility, we correlated each participant’s scene drop values with their mean number of correct list B recalls (i.e., recall of list B items on blocks where they were instructed to recall list B). None of these correlations were significant, and they were all numerically negative (i.e., contextual change, as operationalized by scene drop, led to numerically worse list B recall). We ran this analysis separately on forget blocks (*r*=−0.22,*p*=0.3), on remember blocks (*r*=−0.07,*p*=0.7), and on remember and forget blocks together (*r*=−0.15,*p*=0.5). Thus, our results do not support a model whereby reducing the activation of list A contextual information supports better list B recall.

More generally, one might hypothesize that participants who are particularly adept at forgetting list A by changing their mental context might also be especially good at recalling list B. In fact, however, our data support the opposite conclusion: participants’ overall memories for list A and B were correlated (*r*=0.62,*p*=0.001). This correlation held when we limited the analysis to forget trials only (*r*=0.61,*p*=0.002) and to remember trials only (*r*=0.54,*p*=0.006). In other words, the participants who displayed the strongest behavioral signatures of forgetting list A following a forget instruction were *not* the same participants who displayed the strongest list B recall performance.

## Discussion

We sought to test the hypothesis that participants can intentionally forget information pertaining to previously experienced events by changing their mental context. When participants studied a list of words (list A), we “injected” scene information into their mental context by presenting scene images between the words. We then used pattern classifiers to estimate the level of scene-related activity as participants studied a second list of words (list B), manipulating whether participants were asked to forget (or remember) the list A words prior to studying list B. We found that the levels of scene-related activity measured during list B were reliably lower following a forget instruction than following a remember instruction. Across participants, the magnitude of the decrease in scene activity in response to a forget instruction (“scene drop”) was negatively correlated with participants’ list A recall performance on that block.

We note that our data do not address whether the neural signatures of scene-related activity we tracked are related to individual scene images or a more general sense of being in a context where scenes are present. Our main claims do not require distinguishing between these possibilities; rather, our goal was to test the contextual change hypothesis by asking whether scene-related activity associated with list A decreased more strongly following a forget (versus remember) memory instruction. Our results provide the first neural support for this hypothesis.

### What is ’context?’

*Context* is both one of the most fundamental and most elusive concepts in memory research. Often memory theorists define context by exclusion: in memory experiments, there are the *items* to be remembered and *context*, which reflects everything else represented in the person’s mind during the experiment and critically influences how the items are organized and retrieved from memory. As reviewed by Smith and Vela (2001), context might include information about the external environment, mood, thoughts about recently encountered items, incidental features of the items (e.g. their color and location on the screen), or even other stimuli presented between the items (as in the present study). However, under this definition, *anything* can be context. For example, if we had tested participants’ memories of the scene images in our study, then we might have considered those scene images to be “items” and the studied words to be part of the scenes’ contexts.

Rather than basing our definition of context on what participants are (or are not) asked to remember, we take a more empirical approach of defining context based on the *timescale* of representations; according to this view, slowly drifting mental representations (i.e., representations of thoughts that persist over relatively long time scales) act to contextualize more quickly drifting mental representations (corresponding to the currently presented item) (Manning et al. 2014). The scene representations that we are measuring with our classifier satisfy this timescale-based definition of context, insofar as these representations persist throughout the list A study period and into the list B study period (when scenes are no longer visually present).

Also, crucially, we do not think that scene information is the only kind of context that is present during list A; each participant will have their own idiosyncratic constellation of slowly drifting thoughts. In our study, we focused on the level of scene activity as an indicator of contextual change because we purposefully “injected” scene activity into participants’ mental context during list A, and we have sensitive tools for measuring scene activity with fMRI. If we had a way of identifying and tracking other kinds of context that were present during list A, we would expect to see a similar decrease in these other kinds of contextual activity in response to the forget cue.

### Potential mechanisms

Although our results provide evidence that we can intentionally forget by changing our contextual states, our results do not pin down the underlying mechanism of contextual change. One possibility is that, in response to the forget instruction, participants change their mental contexts by trying to think new thoughts unrelated to the experiment, which then “push out” the old contextual information. For example, Sahakyan and Kelley (2002) showed that asking participants to change their mental context by actively thinking about some other cognitively rich topic (e.g. thinking about what they would like to do if they were invisible) after list A was effective in reducing list A recall. Another possibility is that participants attempt to directly suppress thoughts related to list A, without “adding in” new thoughts; for discussion of this possibility see Anderson and Hanslmayr (2014). Both of these interpretations are consistent with our finding that scene-related activity decreases more following forget (versus remember) instructions. Specifically, the decrease in scene-related activity we observed might be driven by the presence of new thoughts (unrelated to scenes) that push out scene-related activity, or the direct suppression of scene-related thoughts.

Another possible explanation for list-method directed forgetting effects, distinct from the contextual change hypothesis, is selective rehearsal (Bjork 1970). For example, following the remember/forget cue, participants might mentally rehearse both list A and list B words during remember blocks, but only list B words during forget blocks. Note that, under this account, participants would not have any motivation to rehearse the *scenes* from list A during remember blocks (since the scenes are not tested later). However, it is possible that rehearsal of list A words (during list B study) on remember blocks could incidentally trigger retrieval of scene context from list A, thereby resulting in higher levels of measured scene activity during list B study on remember (vs. forget) blocks.

Our study does not specifically rule out selective rehearsal accounts of the effects we observed. However, a number of behavioral studies show that differences in rehearsal strategies are unlikely to account for list-method directed forgetting effects. For example, as mentioned above, list-method directed forgetting effects are absent from standard recognition memory tests (Basden et al. 1993; Bjork and Bjork 2003); one would expect differential rehearsal of list A items to affect performance on both free recall and recognition memory tests. Second, directed forgetting effects are also observed during incidental learning (where no rehearsal is implicated; Geiselman et al., 1983; Sahakyan and Delaney, 2005). Therefore, if rehearsal does play some role in list-method directed forgetting, it is clearly not the entire picture. For further review of the evidence against selective rehearsal accounts of list-method directed forgetting see Sahakyan et al. (2013).

### Relation to prior neural studies of directed forgetting

Whereas the focus in our study was on the evoked response to the forget cue, other recent work has focused on how neural activity during list B study varies as a function of whether participants are given a forget or remember cue. In particular, Hanslmayr et al. (2012) and Bäuml et al. (2008) found EEG desynchronization effects during list B that were larger following forget cues than remember cues. These desynchronization effects were correlated (across subjects) with the behavioral cost of directed forgetting (Bäuml et al. 2008) and were accompanied by an increase in dorsolateral prefrontal cortical (dlPFC) activity (Hanslmayr et al. 2012). Further, stimulating the dlPFC with transcranial magnetic stimulation (TMS) increased the magnitude of these desynchronization effects and led to increased list A forgetting. The authors interpret these findings as showing that active control processes as participants study list B contribute to list A forgetting.

Taken together, our study and these previous studies suggest that the process of shifting one’s mental context has multiple components. Our results show that, after being told to forget list A, participants respond to this cue by changing their mental context; this contextual change process takes place right away, before the start of list B. Speculatively, the desynchronization effect described by Hanslmayr et al. (2012) and Bäuml et al. (2008) may reflect loading new stimulus features (e.g. those that will become associated with list B) into context during list B study.

One last puzzle is how to reconcile our results (showing that scene drop, measured prior to list B study, is associated with impaired list A recall) with other results showing that study of list B items is needed to induce directed forgetting (e.g. Pastötter and Bäuml, 2007, but see Unsworth et al., 2012). One possibility is that participants can reinstate list A context if they are tested immediately after the forget cue, but this recovery becomes more difficult once participants have loaded in a new contextual state that incorporates list B items (Sahakyan et al. 2013; Lehman and Malmberg 2009).

### Concluding remarks

We used a simple list-learning paradigm to elucidate the neural mechanisms underlying how we intentionally forget. Our work highlights the fundamental role that contextual information plays in our ability to organize and retrieve information pertaining to previous experiences, and it provides neural support for the hypothesis that we can forget about our recent past by changing our mental context.
